# Camptothecin-Based Regimens for Treatment of Ewing Sarcoma: Past Studies and Future Directions

**DOI:** 10.1155/2011/957957

**Published:** 2011-04-06

**Authors:** Lars Wagner

**Affiliations:** Division of Pediatric Hematology/Oncology, Cincinnati Children's Hospital Medical Center, University of Cincinnati College of Medicine, MLC 7015, 3333 Burnet Avenue, Cincinnati, OH 45229, USA

## Abstract

New therapies are needed to improve survival for patients with Ewing sarcoma. Over the past decade, camptothecin agents such as topotecan and irinotecan have demonstrated activity against Ewing sarcoma, especially in combination with alkylating agents. Previous studies have shown camptothecin-based combinations to be tolerable outpatient strategies that are attractive for salvage therapy. This paper highlights important issues related to drug dosing, schedule of administration, pharmacokinetics, toxicity, and activity of commonly used camptothecin-based regimens. Also discussed are strategies for incorporating these regimens into therapy for newly diagnosed patients, including several potential possibilities for combination with targeted agents.

## 1. Introduction

Current treatments using interval compression of multiagent chemotherapy combined with surgery and/or radiation can now cure the majority of patients with localized Ewing sarcoma [1]. However, outcomes are much worse for patients who develop disease recurrence after initial therapy, or who have metastases at diagnosis. For example, fewer than 20% of patients with relapsed Ewing sarcoma are likely to be long-term survivors [2], and patients who present with metastatic bone and/or bone marrow disease at initial diagnosis share a similarly poor prognosis [3]. New therapeutic approaches are clearly necessary to improve overall survival for these patients. 

Chemotherapy for Ewing sarcoma has historically consisted of alkylating agents and anthracyclines. Two modifications of this backbone have led to significant improvements in outcome and have helped to define the current standard of care. First, the addition of cassettes of ifosfamide and etoposide onto the 3-drug combination of vincristine, doxorubicin, and cyclophosphamide has improved survival for patients with localized disease [4]. Building on these results, Womer et al. have shown that compression of the interval between treatment courses from 3 weeks to 2 weeks provides additional improvement in 3-year event-free survival, which is now up to 76% for nonmetastatic patients [1]. Since further modification of this 5-drug regimen seems unlikely to produce additional benefits [5], future regimens will likely need to incorporate new drugs in order to continue progress in the field.

Camptothecin agents have been evaluated in Ewing sarcoma patients for the last decade. This class of agents is attractive because of commercial availability, modest single-agent activity, and demonstrated tolerability and synergy with alkylating agents. Camptothecins exert cytotoxicity by stabilizing the covalent complex between DNA and topoisomerase I, the enzyme which relieves torsional strain of DNA. This stabilization process prevents religation of DNA, and the ensuing collision of the stabilized complex with the advancing replication fork results in double strand breaks and cell death. Preclinical studies have suggested that activity of camptothecins is greatest when given in combination with alkylating agents [6]. Mechanistically, DNA damage from alkylators may increase the importance of topoisomerase I for cell repair, thus rendering tumor cells more sensitive to topoisomerase I inhibition [7]. This activity is also enhanced in preclinical experiments when protracted, low-dose administration is used compared to single large doses [8], which is consistent with the S-phase-specific mechanism of action.

This paper summarizes past Ewing sarcoma clinical trials of the two most commonly used camptothecins, topotecan, and irinotecan. Emphasis is placed on differences in their toxicity profiles, schedules, and routes of administration, and partnering alkylating agents. Finally, an outline is provided regarding potential future directions in which these agents may be further developed.

## 2. Topotecan

### 2.1. Dosing and Schedule of Administration

Topotecan was the first camptothecin to be tested in Phase II trials against Ewing sarcoma. Various schedules of administration have been evaluated, including continuous infusions lasting from 72 hours [9] to 21 days [10]. Although continuous exposure is intuitively attractive for maximizing activity of S-phase-specific drugs, this approach may actually downregulate free topoisomerase I and lead to drug resistance [11]. Therefore, repeated administration on a protracted schedule may be more beneficial [8]. The most commonly used strategy is to administer 5-day courses of topotecan as a daily 30-minute infusion [12]. Dosages studied have ranged from 0.75–3 mg/m^2^/day, depending on whether topotecan is given as a single agent or in combination with other chemotherapeutics. As discussed below, the greatest activity for topotecan against Ewing sarcoma has been in combination with cyclophosphamide, in which both drugs are given daily for five days (i.e., d × 5 schedule) [13, 14]. Courses generally are repeated every 21 days, and timely administration is facilitated by the concurrent use of filgrastim.

### 2.2. Pharmacokinetics

There is significant interpatient variability in topotecan pharmacokinetics, with 10-fold differences in systemic clearance of topotecan lactone, which is the form of topotecan with greatest antitumor activity [15]. This has led investigators at St. Jude Children's Research Hospital to explore targeting the topotecan dose of individual patients to achieve a prespecified systemic exposure that correlated with activity in mouse tumor models. In one study, administration of a standard topotecan dose resulted in achieving the desired systemic exposure in only 53% of patients. However, when patients underwent real-time pharmacokinetic testing with the first dose and then had subsequent doses modified based on these results, 72% of patients finished the course within the targeted drug exposure range [15]. This experience, coupled with data from other clinical trials, allowed for the development of a population pharmacokinetic model which used factors such as body surface area, glomerular filtration rate, and age to help predict up to 76% of interpatient variability in topotecan clearance [16]. However, to date, neither use of this model nor complicated pharmacokinetic targeting has been employed for treatment of Ewing sarcoma patients, and it remains unclear to what extent the variance in systemic exposure affects overall activity of camptothecin agents, particularly when used in combination with other drugs.

### 2.3. Route of Administration

Intravenous administration is the most common method of topotecan administration; however, it is both costly and inconvenient, particularly when protracted schedules are used. These factors have led to the study of oral topotecan administration. Daw et al. reported that the oral bioavailability of the intravenous preparation given orally was 0.27 and 0.36 when administered in a gelatin capsule [17]. At the maximum tolerated dose (MTD) of 1.8 mg/m^2^ given daily for five days for two consecutive weeks (i.e., d × 5 × 2), systemic exposures were similar to those required to achieve responses in mouse models. Further studies have tested oral topotecan in combination with daily oral cyclophosphamide [18], and with both drugs together with gefitinib in a strategy described in greater detail below [19]. Capsule formulation of topotecan is now approved by the FDA for treatment of relapsed lung cancer, based in part on encouraging results from an adult Phase III trial showing similar activity and tolerability when oral dosing of 2.3 mg/m^2^/day × 5 was compared to 1.5 mg/m^2^/day × 5 given intravenously [20]. There have been no reported Phase II trials of oral topotecan in Ewing sarcoma patients to date.

### 2.4. Toxicities

The primary dose-limiting toxicity of topotecan is myelosuppression. In single-agent trials, nonhematologic grade 3-4 toxicities such as nausea or dehydration occur in less than 10% of patients [9, 10]. Rare events such as fever and mucositis have also been reported to occur with single-agent topotecan. With more protracted schedules in which drug is administered for 5 days for two consecutive weeks (i.e., d × 5 × 2 schedule), diarrhea also may be a prominent complication [17].

When topotecan is given in combination with cyclophosphamide at the recommended Phase II dose (topotecan 0.75 mg/m^2^/day × 5; cyclophosphamide 250 mg/m^2^/day × 5), myelosuppression remains the predominant toxicity. In the largest reported Phase II experience, 163 (53%) of 307 courses given to 83 evaluable pediatric patients with recurrent solid tumors resulted in grade 3-4 neutropenia, despite all patients receiving prophylactic filgrastim [13]. However, no infectious deaths were observed, and Grades 3-4 infections were reported in only 11% of courses. Thrombocytopenia reaching Grades 3-4 was observed in 44% of all courses.

### 2.5. Activity against Ewing Sarcoma

Although a sustained complete response was reported in one of 5 Ewing sarcoma xenograft models treated with single-agent topotecan [21], clinical trials of topotecan by itself for recurrent Ewing sarcoma have been relatively disappointing. For example, the response rates for relapsed/refractory Ewing sarcoma patients treated on three Phase II studies using various doses and schedules were 4%, 8%, and 10% [9, 10, 12]. However, combination with cyclophosphamide markedly improves the response rate in this patient population, with two Phase II studies showing response rates of 33% and 35% [13, 14]. In addition, approximately one-third of patients also had stable disease or minor/mixed responses. The success of this combination has resulted in its frequent use for salvage therapy for patients in first relapse. 

A pilot study exploring higher doses of these agents was recently reported by Kushner et al., using cyclophosphamide 4200 mg/m^2^ by 48-hour infusion together with topotecan 6 mg/m^2^ by 72-hour infusion [22]. As expected, myelosuppression was more frequent and severe, although retreatment was possible within 28 days. The limited number of Ewing sarcoma patients studied precludes assessment of whether higher doses improve activity for this tumor type. In addition, because alkylating agents may have significant single agent activity [23], determination of the relative contribution of topotecan for treating Ewing sarcoma is difficult. Details of key clinical trials using topotecan for Ewing sarcoma are summarized in Table 1.

## 3. Irinotecan

### 3.1. Dosing and Schedule of Administration

As with topotecan, scheduling of irinotecan administration for pediatric solid tumors has tried to capitalize on the S-phase-specific mechanism of action, with more protracted schedules having greater preclinical activity [8]. Many early trials used the d × 5 × 2 schedule modeled after a pediatric Phase I trial of intravenous irinotecan reported by Furman et al., which established the MTD of 20 mg/m^2^/day [24]. However, when compared directly with a d × 5 schedule using a dose of 50 mg/m^2^/day × 5, there was no difference in response rates or incidence of grade 3-4 toxicities in patients with recurrent rhabdomyosarcoma who were receiving intravenous irinotecan together with vincristine as a 6-week upfront window [25]. This is the only pediatric trial which compared two schedules in a randomized fashion, and the results are similar to comparisons of different schedules in colon cancer patients [26]. Although it is possible that the more protracted d × 5 × 2 schedule may have greater benefit with diseases other than rhabdomyosarcoma, it is likely that these results will be extrapolated to future Ewing sarcoma trials using irinotecan, given the improved patient convenience and decreased costs of shorter treatment schedules.

### 3.2. Pharmacokinetics

Similar to topotecan, there is up to one log variability in drug exposures between patients receiving irinotecan [24]. Irinotecan is functionally a prodrug, which is converted by endogenous carboxylesterases to the active topoisomerase I poison SN-38. In adults receiving large single doses of irinotecan every 1–3 weeks, metabolism is significantly affected by polymorphisms in *UGT1A1*, which controls inactivation of SN-38 through the process of glucuronidation [27]. For the approximately 10% of patients who are homozygous for the UGT1A1*28 genotype, the risk of toxicity is substantial, resulting in an FDA recommendation for dose reduction in starting doses for these patients [28]. Importantly, this polymorphism has had less impact in pediatric patients receiving protracted irinotecan, as increased toxicity has not been associated with UGT1A1*28 in over 180 patients in 4 clinical trials [29–32]. To date, no genetic factors have been identified to reliably predict toxicity in children receiving protracted irinotecan.

### 3.3. Route of Administration

As with topotecan, there has been interest in oral administration of irinotecan as a way to improve convenience and reduce cost. Three studies of oral irinotecan have been completed and have demonstrated that administration of the intravenous preparation mixed in cran-grape juice, despite its bitter taste, is indeed feasible over multiple courses in pediatric patients [31–34]. Cost savings have been estimated to be up to fivefold when compared to intravenous administration [34]. Most importantly, mean SN-38 exposures for patients treated at the MTD of 60 mg/m^2^/day on the d × 5 × 2 schedule are similar to those from patients receiving intravenous irinotecan at the MTD of 20 mg/m^2^/day [24, 32, 33]. Analogously, exposures from patients receiving oral irinotecan at 90 mg/m^2^/day correlate with those receiving intravenous irinotecan at 50 mg/m^2^/day [31]. These studies show that for patients for whom oral administration is suitable, this strategy can greatly reduce costs and improve convenience, particularly when partner drugs such as temozolomide are also given orally.

### 3.4. Toxicities

The dose-limiting toxicity of irinotecan is dependent on the schedule used. For shorter administration schedules, such as the single doses given every 1–3 weeks in most adult studies, myelosuppression is dose limiting [26]. In contrast, late-onset diarrhea and abdominal cramping are dose limiting when irinotecan is given on the protracted d × 5 × 2 schedule [24]. For the intermediate d × 5 schedule, both myelosuppression and diarrhea can be seen, although both toxicities are generally modest and manageable [29]. This favorable toxicity profile has allowed for combination with agents like temozolomide, in which the toxicity is primarily hematologic. In fact, the temozolomide + irinotecan combination can generally be given in three week courses without the routine use of filgrastim support [35, 36].

Although severe diarrhea from protracted irinotecan occurs in ≤20% of patients, such morbidity can impact compliance [37], even when the tumor is responding to treatment [38]. Recent efforts to reduce irinotecan-associated diarrhea in pediatric patients have included the use of activated charcoal to reduce intestinal absorption of the active metabolite SN-38 [39], as well as prophylactic cephalosporins [33, 40]. Antibiotics work by reducing the intestinal colonization of Gram-negative aerobic bacteria, which produce the glucuronidase enzyme that converts inactivated SN-38-glucuronide back to active SN-38, causing direct injury to the colon. Cephalosporins such as cefixime have been helpful in allowing 50% higher doses of oral irinotecan to be tolerable, resulting in improved systemic exposures [33]. Cephalosporin prophylaxis has been used in all three reported trials of oral irinotecan, and also has been used in patients who have experienced severe gastrointestinal toxicity with previous courses of intravenous irinotecan [41]. However, use of antibiotics is not required for all patients receiving standard doses of intravenous irinotecan, and prolonged cephalosporins may be deleterious if given unnecessarily. When used, cephalosporins are started at least two days prior to chemotherapy and continued for at least three days after chemotherapy. Therefore, patients receiving 5-day courses of irinotecan can be treated with a 10-day course of antibiotics [31], while patients receiving longer schedules of irinotecan are often continued indefinitely on antibiotics [32]. 

Although most patients receiving irinotecan and temozolomide do not have significant cumulative toxicity, a recent trial using vincristine, oral irinotecan, and temozolomide (VOIT regimen) showed a subset of patients who experienced fatigue, anorexia, and weight loss that resulted in removal from study [31]. This finding has not been reported in previous studies, but does underscore the point that this regimen may not be well tolerated over multiple courses by all patients.

### 3.5. Activity against Ewing Sarcoma

Although not formally tested against Ewing sarcoma models, there have been reports of complete sustained responses with single-agent irinotecan in xenograft models of primitive neuroectodermal tumor (PNET), which is clinically similar to Ewing sarcoma [42]. Building on this preclinical data, Bisogno et al. reported a response rate of 38% in 13 patients with recurrent/refractory extraosseous PNET who were treated with protracted intravenous irinotecan given on a d × 5 × 2 schedule [38]. However, no responses were seen in 16 patients with recurrent/refractory PNET/ES in a Children's Oncology Group Phase II trial using intravenous irinotecan 50 mg/m^2^/day × 5 [29]. 

 It is likely that the greatest antitumor activity of irinotecan will be in combination with other drugs, such as the methylating agent temozolomide. Houghton and colleagues have demonstrated a schedule-dependent synergy between temozolomide and irinotecan, in which methyl groups placed on DNA by temozolomide cause recruitment of topoisomerase I, thus potentiating the cytotoxic effects of irinotecan given at least one hour later [6, 7, 43]. This two-drug combination has now been studied in three trials, with reported response rates of 29%, 64%, and 63% [35, 36, 44]. Collectively, the cumulative results show partial or complete responses in 32 (55%) of 58 patients with recurrent/refractory Ewing sarcoma who have received 5-day courses of temozolomide combined with intravenous irinotecan given on the d × 5 × 2 schedule. Based on this data, temozolomide + irinotecan, like cyclophosphamide + topotecan, has become a widely used regimen for patients with relapsed or refractory Ewing sarcoma. Interestingly, the mechanisms of resistance to these camptothecin agents appear to be different [45], and responses have been reported with one regimen even when disease progression has been noted with the other [35].

Based on the established role of vincristine in treating Ewing sarcoma, as well as the synergy observed in rhabdomyosarcoma patients who are treated with vincristine + irinotecan [41], more recent trials have added vincristine onto the backbone of temozolomide + irinotecan [31, 46]. Use of vincristine may be particularly attractive for newly diagnosed patients, for whom use of a known active agent like vincristine may help reduce the rate of early disease progression when using an upfront-window design [41]. This addition does not seem to significantly change the toxicity profile, although comparison studies with and without vincristine have not been performed. Details of key clinical trials using irinotecan for relapsed/refractory Ewing sarcoma are provided in Table 2.

## 4. Comparison of Topotecan-Based versus Irinotecan-Based Regimens

Because of the relatively small number of patients with recurrent Ewing sarcoma, randomized trials directly comparing regimens have not been performed.  Table 3 summarizes available information for commonly used salvage therapies, highlighting toxicities, drug administration factors, and response rates. The prioritization of salvage regimens depends on several factors, including individual patient considerations. Temozolomide + irinotecan is generally less myelosuppressive than cyclophosphamide + topotecan and does not usually require myeloid growth factor support. However, some patients may develop diarrhea, fatigue, or weight loss that becomes problematic. Although both regimens can potentially be given orally, there is a larger published pediatric experience with oral irinotecan (summarized in [34]). Home administration of chemotherapy, whether intravenously or oral, is often attractive for patients, and has been well described with temozolomide + irinotecan [35]. Home administration also helps reduce cost, which can be considerable if multiple courses are administered. At our institution, patient drug costs alone for an individual with body surface area of 1.5 m^2^ receiving a standard 5-day course of cyclophosphamide + topotecan + pegfilgrastim are $21,966 (USD), compared to a standard 5-day course of temozolomide + oral irinotecan costing $17,599. The total number of courses given to patients with relapsed disease is variable and depends on both response and tolerability. Extended treatment using cyclophosphamide + topotecan (up to 11 courses) and temozolomide + irinotecan (up to 20 courses) has been described, but the benefits of such protracted therapy are unclear. Relapsed patients may also benefit from local control measures such as radiotherapy [48], which in most situations can be administered concurrently with either regimen.

## 5. Further Development of Camptothecins in Treatment of Ewing Sarcoma

There are two general ways that camptothecin regimens can be incorporated into frontline therapies. The first is to build on standard 5-drug therapy by inserting cassettes of either cyclophosphamide + topotecan, or temozolomide + irinotecan + vincristine. Based on the success of administering standard 5-drug treatment in two-week intervals [1], the Children's Oncology Group (COG) has performed a feasibility trial examining whether cyclophosphamide + topotecan may also be given in intervals shorter than the typical 3 weeks. Published results from this trial are currently pending; however, the Children's Oncology Group has now opened a randomized Phase III trial in which patients with newly diagnosed localized Ewing sarcoma may receive cassettes of vincristine, cyclophosphamide, and topotecan in 2-week intervals interspersed in the standard 5-drug regimen (http://clinicaltrials.gov/#NCT01231906). 

The second strategy is to use camptothecin regimens as a therapeutic backbone on which to add targeted therapies. Several candidate agents have been evaluated either in clinical trials or preclinical experiments and are discussed below.

## 6. Combination Strategies

### 6.1. Bevacizumab

Bevacizumab is a monoclonal antibody directed against vascular endothelial growth factor, and targets the newly developing blood vessels necessary for sustaining solid tumors. Although originally thought to have antitumor activity by “choking off” the blood supply to tumors, the primary effect of bevacizumab may actually be related to normalization of the tumor-associated vasculature by pruning off immature or poorly formed blood vessels, thereby improving distribution of chemotherapy [49]. This principle was demonstrated in preclinical experiments by Dickson et al., in which intratumoral topotecan concentrations improved following pretreatment with bevacizumab, which corresponded to better antitumor activity [50]. Given the long half-life of bevacizumab, and the demonstration that intratumoral perfusion peaks 2 days after administration and returns to baseline within 7 days, combination with protracted courses of camptothecins seems reasonable. The COG has recently finished a pilot study in which bevacizumab was added to the backbone of vincristine, cyclophosphamide, and topotecan for patients with relapsed/refractory Ewing sarcoma [51]. This combination was feasible, and future studies are ongoing, such as an institutional trial at Cincinnati Children's Hospital which combines bevacizumab with the VOIT regimen (vincristine, oral irinotecan, and temozolomide) (http://clinicaltrials.gov/#NCT00786669).

### 6.2. IGF-1R Antibodies

Signaling through the insulin-like growth factor type I receptor (IGF-1R) is critical for the growth and maintenance of Ewing sarcoma cells, and its inhibition offers an attractive new target for therapy (reviewed in [52]). IGF-1R also has important effects on apoptosis and response to DNA damage. Wang et al. have shown that the concomitant use of an IGF-1R antibody together with irinotecan produced additive effects and resulted in a 93% reduction in tumor volume in a mouse model of thyroid carcinoma [53]. Clinical trials of anti-IGF-1R antibodies have already demonstrated single-agent activity against relapsed Ewing sarcoma [54], with one of the largest showing 2 (13%) of 16 patients having objective responses, with another 8 (50%) having stable disease for at least 4 months [55]. The COG is now using the IMC-A12 antibody together with multiagent chemotherapy in a study for metastatic rhabdomyosarcoma (http://clinicaltrials.gov/#NCT01055314), and combination strategies for Ewing sarcoma are in early development (http://clinicaltrials.gov/#NCT01182883).

### 6.3. Gefitinib

Gefitinib is a small molecule inhibitor of the epidermal growth factor receptor and is used clinically for treatment of lung cancer. Gefitinib competes with camptothecins at the level of the ABCG2 drug efflux pump, causing inhibition of the pump without directly serving as a substrate [56]. This allows gefitinib to reverse irinotecan resistance *in vitro* [57], even in cell lines that lack expression of the epidermal growth factor receptor. Since ABCG2 is expressed in normal small intestine, administration of gefitinib also improves by threefold the oral bioavailability of irinotecan in mice [56]. This data has led to recent trials of gefitinib + oral irinotecan for refractory pediatric solid tumors [58], as well as gefitinib + oral topotecan and cyclophosphamide in children with relapsed neuroblastoma [19].

### 6.4. mTOR Inhibitors

Like gefitinib, the mTOR inhibitor rapamycin inhibits ABCG2 without being a substrate of this pump. Accordingly, rapamycin has effectively reversed *in vitro* resistance to topotecan [59]. Rapamycin is also synergistic with irinotecan in mouse models of colon cancer, perhaps through inhibition of hypoxia-inducible factor-1 alpha [60]. mTOR inhibitors have had some modest single-agent effects against Ewing sarcoma xenografts [61], and a recently opened COG Phase I trial will examine the combination of temozolomide and irinotecan together with the once-weekly mTOR inhibitor temsirolimus (http://clinicaltrials.gov/#NCT01141244).

### 6.5. PARP Inhibitors

Poly (ADP-ribose) polymerase (PARP) is a key mediator of DNA base excision repair and recovery from DNA strand breaks and protect cells from the toxicity of various chemotherapy agents. Inhibitors of PARP can effectively enhance the *in vitro* and *in vivo* activity of topotecan, irinotecan, and temozolomide against colorectal and lung cancer cell lines and xenograft models [62]. Interestingly, Tentori et al. demonstrated that PARP inhibition not only increased the activity of temozolomide + irinotecan against tumor xenografts, but also reduced intestinal injury and diarrhea in mouse models, thus offering the possibility of improved activity with less toxicity [63].

### 6.6. New Camptothecins in Development

EZN-2208 is a novel polymer-drug conjugate which links the active metabolite of irinotecan (SN-38) with a multiarm polyethylene glycol. This allows for intravenous administration once every three weeks, with sustained SN-38 exposures that are superior to those seen with protracted irinotecan [64]. In experimental models, activity has been seen even in tumors resistant to irinotecan [65]. Pediatric trials of EZN-2208 are set to begin soon.

## 7. Conclusions

Considerable progress has been made in evaluating camptothecin agents for treatment of Ewing sarcoma, and both topotecan and irinotecan clearly are active when combined with alkylating agents. These drugs have several similarities, including superior activity when given on protracted schedules, wide interpatient variability in pharmacokinetics, and the potential for oral administration. Both cyclophosphamide + topotecan and temozolomide + irinotecan are now widely used for patients with recurrent tumors and offer a good chance of response or disease stabilization. Decisions regarding which regimen to use are often based on toxicity and drug administration issues. The activity and tolerability seen with both regimens assures further exploration in upcoming trials, either as cassettes which are incorporated into standard five-drug regimens or as therapeutic backbones on which to add targeted agents.

## Figures and Tables

**Table 1:** Summary of selected studies using topotecan-based therapy for patients with relapsed/refractory Ewing sarcoma.

Author [Ref]	*N*	Topotecan dose and schedule	Other drugs	Response Rate (complete + partial)	Comments
Hawkins et al. [10]	20	0.3 mg/m^2^/d for 21-day continuous infusion	—	10%	
Blaney et al. [9]	3	1–1.3 mg/m^2^/d for 72-hour continuous infusion	—	4%	
Bernstein et al. [12]	36	2 mg/m^2^/d × 5	—	8%	Given as upfront window for 6 weeks in newly diagnosed metastatic patients
Bernstein et al. [12]	37	0.75 mg/m^2^/d × 5	CPM 250 mg/m^2^/d × 5	57%*	Given as upfront window for 6 weeks in newly diagnosed metastatic patients
Saylors et al. [13]	17	0.75 mg/m^2^/d × 5	CPM 250 mg/m^2^/d × 5	35%	50% of patients received at least 6 courses before progression
Hunold et al. [14]	49	0.75 mg/m^2^/d × 5	CPM 250 mg/m^2^/d × 5	33%	Additional 26% with stable disease

*In newly diagnosed patients with metastatic disease; CPM, cyclophosphamide.

**Table 2:** Summary of studies using irinotecan-based therapy for patients with relapsed/refractory Ewing sarcoma.

Author [Ref]	*N*	IRN dose and schedule	Route of IRN administration	TEM dose	Other drugs	Response rate (complete + partial)	Comments
Bisogno et al. [38]	13	20 mg/m^2^/d × 5 × 2	IV	—	—	38%	Study limited to soft tissue PNET patients
Cosetti et al. [37]	3	20 mg/m^2^/d × 5 × 2	IV	—	—	0	
Bomgaars et al. [29]	16	50 mg/m^2^/d × 5	IV	—	—	0	
Casey et al. [36]	20	10 mg/m^2^/d × 5 × 2	IV	100 mg/m^2^/d × 5	—	63%	Median TTP = 8.3 months
Wagner et al. [35]	14	10–20 mg/m^2^/d × 5 × 2	IV	100 mg/m^2^/d × 5	—	29%	50% of patients received at least 6 courses before progression
Anderson et al. [44]	25	10 mg/m^2^/d × 5 × 2	IV	100 mg/m^2^/d × 5	—	60%	Median TTP = 5.5 months
Wagner et al. [31]	5	35–90 mg/m^2^/day	PO	100–150 mg/m^2^/d × 5	VCR	40%*	Phase I trial using different doses and schedules
McNall-Knapp et al. [46]	1	15 mg/m^2^/d × 5 × 2	IV	100 mg/m^2^/d × 5	VCR	100% (*n* = 1)	

*Two of five patients had initial responses, but came off study prior to repeat imaging VCR, vincristine; TEM, temozolomide; IRN, irinotecan; TTP, time to progression.

**Table 3:** Comparison of commonly used regimens for recurrent Ewing sarcoma.

	Cyclophosphamide + topotecan	Temozolomide + irinotecan	High-dose ifosfamide	Ifosfamide + carboplatin + etoposide
Number of patients	66	58	37	22
Author (Ref)	Saylors et al. [13] Hunold et al. [14]	Casey et al. [36] Wagner et al. [35] Anderson et al. [44]	Ferrari et al. [23]	Van Winkle et al. [47]
Primary toxicity	Myelosuppression	Gastrointestinal	Myelosuppression	Myelosuppression
Alopecia	Yes	Uncommon	Yes	Yes
Myeloid growth factor	Yes	Rarely	Yes	Yes
IV access required	Generally	Optional if administered orally	Yes	Yes
Home administration	Uncommon	Common	No	No
Response rates (complete + partial)	33–35%	29–63%	34%	48%
